# Two-stage prediction model for in-hospital mortality of patients with influenza infection

**DOI:** 10.1186/s12879-021-06169-6

**Published:** 2021-05-19

**Authors:** Chan-Wa Cheong, Chien-Lin Chen, Chih-Huang Li, Chen-June Seak, Hsiao-Jung Tseng, Kuang-Hung Hsu, Chip-Jin Ng, Cheng-Yu Chien

**Affiliations:** 1 Department of Emergency Medicine, Chang Gung Memorial Hospital, Linkou and College of Medicine, Chang Gung University, Taoyuan, Taiwan; 2 Department of Emergency Medicine, New Taipei Municipal Tucheng Hospital, New Taipei City, Taiwan; 3 Biostatistical Unit, Clinical Trial Center, Chang Gung Memorial Hospital, Linkou, Taiwan; 4 Laboratory for Epidemiology, Chang Gung University, Kwei-Shan, Taiwan; 5 Department of Emergency Medicine, Ton-Yen General Hospital, Zhubei, Taiwan; 6 Graduate Institute of Business and Management, Chang Gung University, Kwei-Shan, Taiwan

## Abstract

**Background:**

Infleunza is a challenging issue in public health. The mortality and morbidity associated with epidemic and pandemic influenza puts a heavy burden on health care system. Most patients with influenza can be treated on an outpatient basis but some required critical care. It is crucial for frontline physicians to stratify influenza patients by level of risk. Therefore, this study aimed to create a prediction model for critical care and in-hospital mortality.

**Methods:**

This retrospective cohort study extracted data from the Chang Gung Research Database. This study included the patients who were diagnosed with influenza between 2010 and 2016. The primary outcome of this study was critical illness. The secondary analysis was to predict in-hospital mortality. A two-stage-modeling method was developed to predict hospital mortality. We constructed a multiple logistic regression model to predict the outcome of critical illness in the first stage, then S1 score were calculated. In the second stage, we used the S1 score and other data to construct a backward multiple logistic regression model. The area under the receiver operating curve was used to assess the predictive value of the model.

**Results:**

In the present study, 1680 patients met the inclusion criteria. The overall ICU admission and in-hospital mortality was 10.36% (174 patients) and 4.29% (72 patients), respectively. In stage I analysis, hypothermia (OR = 1.92), tachypnea (OR = 4.94), lower systolic blood pressure (OR = 2.35), diabetes mellitus (OR = 1.87), leukocytosis (OR = 2.22), leukopenia (OR = 2.70), and a high percentage of segmented neutrophils (OR = 2.10) were associated with ICU admission. Bandemia had the highest odds ratio in the Stage I model (OR = 5.43). In stage II analysis, C-reactive protein (OR = 1.01), blood urea nitrogen (OR = 1.02) and stage I model’s S1 score were assocaited with in-hospital mortality. The area under the curve for the stage I and II model was 0.889 and 0.766, respectively.

**Conclusions:**

The two-stage model is a efficient risk-stratification tool for predicting critical illness and mortailty. The model may be an optional tool other than qSOFA and SIRS criteria.

**Supplementary Information:**

The online version contains supplementary material available at 10.1186/s12879-021-06169-6.

## Background

Influenza has a long history worldwide, but remains a challenging issue in public health. Epidemic waves of seasonal influenza affect health institutes and governments every year. A modeling study investigating the number of influenza-associated respiratory deaths that occurred globally between 1999 and 2015 estimated that 291,243–645,832 respiratory deaths associated with seasonal influenza occurred each year (4.0–8.8 per 100,000 individuals). Mortality rates were highest among people aged 75 years or older (51.3–99.4 per 100,000 individuals) [1]. Influenza pandemics in 1918, 1957, 1968, 1977 and 2009 had catastrophic effects on the medical system and caused many deaths. In 1918, a pandemic caused by the virus H1N1 led to more than 50 million deaths worldwide [2]. In a 2009 pandemic caused by H1N1pdm09, the World Health Organization reported a global total of 18,449 laboratory-confirmed deaths by 1 August 2010 [3]. A modeling study implied that the true burden of mortality from H1N1pdm09 during the 2009 pandemic was probably even higher, with the authors estimating that respiratory mortality was about ten times higher than laboratory-confirmed mortality [4]. The mortality and morbidity associated with epidemic and pandemic influenza puts a heavy burden on hospitals, long-term care units, community clinics, and national health organizations by increasing admission rates. In addition, influenza has a major socioeconomic impact because it causes high levels of worker absenteeism and productivity loss. In the USA, the total economic burden of the 2003 influenza epidemic was US$87.1 billion and the annual burden per capita ranged from US$92 to US$299 [5]. In South Africa, the mean annual economic burden of influenza was estimated at US$270.5 million, while the mean annual burden per capita was US$5.1. The cost per capita in South Africa was lower than it was in European countries and the USA, but was similar to costs per capita in middle-income Asian countries [6].

Vaccines have been developed to reduce influenza infections and lessen the burden of influenza. The WHO recommends that four key populations be prioritized for influenza vaccination: pregnant women, children younger than 5 years of age, people aged 65 years and older, and individuals with underlying health conditions [7]. Vaccines are deployed by many countries each year. However, the protective capacity of a vaccine is affected by the degree of antigenic match between the vaccine and the circulating influenza strains. A vaccine may only provide 50–70% protection, especially in elderly people and people with chronic diseases [2]. This means that some populations are at risk for influenza infection even when a vaccination program is in place [2].

Most patients with mild influenza can be treated on an outpatient basis. Patients with severe influenza, in contrast, require hospitalization or even admission to intensive care units [8]. Influenza can lead to pneumonia, encephalitis, myocarditis, rhabdomyolysis, acute respiratory distress syndrome, and other severe complications, especially among young children (less than 5 years old), elderly people (older then 65 years old), and immunocompromised people [9].

It is crucial for frontline medical units, especially emergency departments (EDs) and urgent care units, to stratify patients with influenza infection by level of risk. Accurate identification of patients who can be safely discharged and patients who need to be admitted to hospital is important from both clinical and economic perspectives. Some studies have attempted to evaluate the efficacy of various tools for predicting risk of mortality in patients with influenza infection. The predictive tools assessed in these studies consider systemic inflammatory response syndrome (SIRS), quick Sequential Organ Failure Assessment scores (qSOFA), and clinical parameters like sex, age, triage category and underlying comorbidities [10–14]. In a single-center, retrospective cohort study conducted in Taiwan, Chu et al. reported that the area under the receiver operating characteristic curve (AUROC) of a qSOFA model for predicting in-hospital mortality in influenza patients was 0.864 [12]. The AUROC of a SIRS model was 0.786 [12]. However, there are discrepancies between the results of the various studies. There is no consensus that one tool is better than the others. Moreover, the primary outcome of the studies investigating the prognostic accuracy of qSOFA and SIRS is in-hospital mortality or 30-day mortality. In practice, frontline clinicians want to identify influenza patients who need admission or critical care in addition to patients who are at higher risk of mortality. Therefore, this study aimed to identify risk factors for needing critical care and to create a prediction model for critical care and in-hospital mortality.

## Materials and methods

### Data sources

We extracted data from the Chang Gung Research Database (CGRD) for this registry-based, retrospective cohort study. The CGRD is a de-identified database derived from the original electronic medical records of Chang Gung Memorial Hospital (CGMH), which is the largest hospital system in Taiwan. It is comprised of seven medical institutes and has 10,070 beds. In 2015, there were over 8,500,000 visits to the outpatient departments of the CGMH and 500,000 visits to the EDs [15]. The CGRD provided all the information required for our study, including patient vital signs, demographic details, laboratory test results, radiology reports, diagnoses, medical history, prescriptions, ED dispositions, and final dispositions of hospitalization and daily medical records. This eliminated the need to extract data manually from electronic medical records or charts. Diagnoses were made and recorded using ICD-10 codes. (For more information on diagnostic categories and clinical data, please refer to Supplementary Tables 1 and 2).

To ensure patients’ privacy, the hospital identification numbers of all patients were encrypted. Data are requested from CGRD via a formal query that is approved by an institutional committee and executed by an analyst. This study was based on a protocol approved by the institutional review board of Chang Gung Memorial Hospital. (201901325B0D001).

### Study cohort

**Fig. 1:**
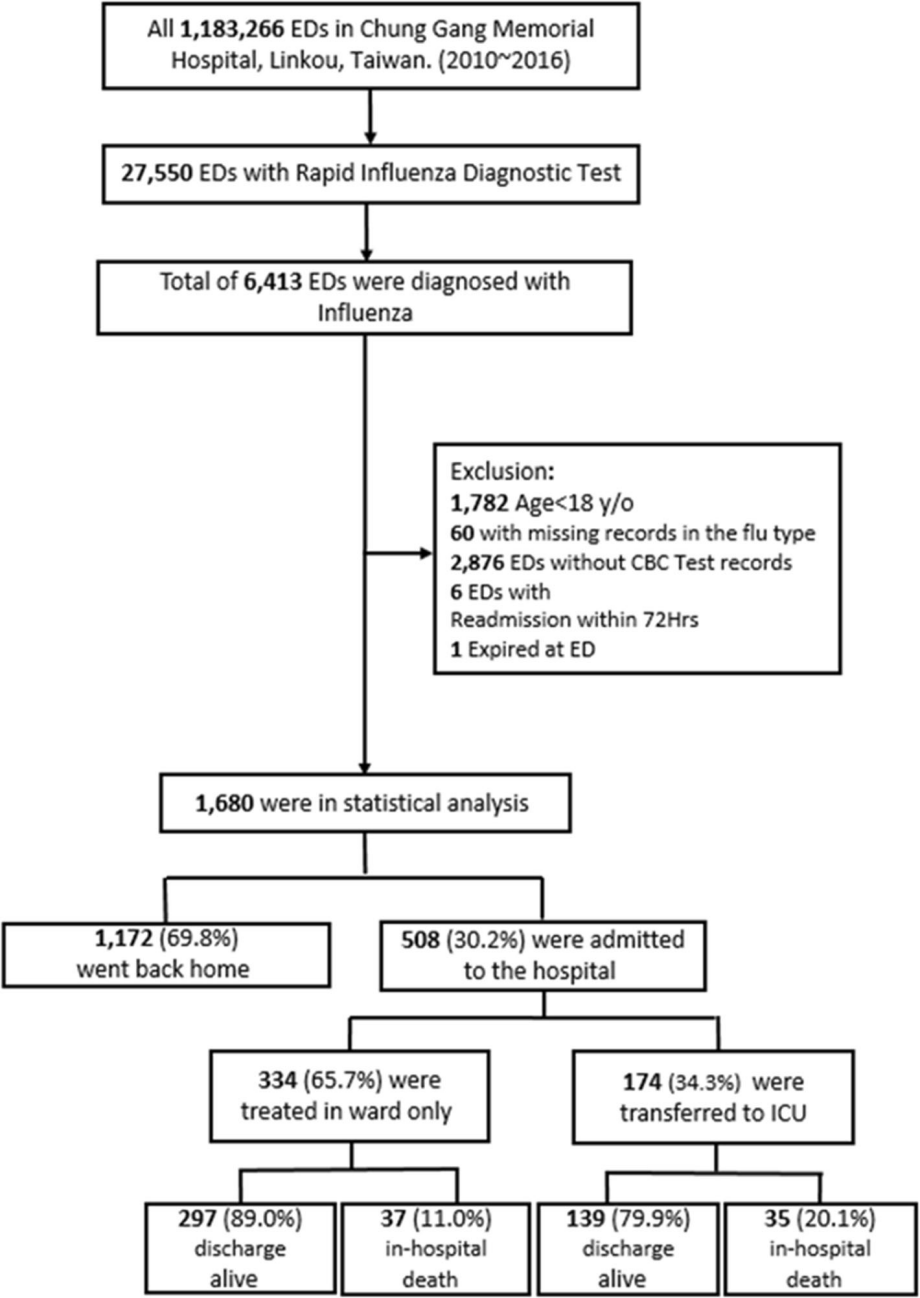
Flow chart of data extraction from Chang Gung Research Database (CGRD)

Between 2010 and 2016, a total of 6413 ED patients were diagnosed with influenza at the CGMH Linkou branch (ICD-10 codes: J09, J10, J11). We excluded patients who were under 18 years of age, had an unidentified virus type, or did not receive a complete blood count test. Ultimately, 1680 ED patients were included in the sample used for data analysis. If a patient was readmitted within 72 h, the admissions were treated as a single episode. We extracted these patients’ laboratory data and clinical outcomes from electronic medical records in the CGRD system (Fig. 1).

### Outcome assessment

The primary outcome of this study was critical illness, which was defined as admission to the ICU or death from severe complicated influenza. The secondary analysis classified influenza patients according to cause of hospital admission and aimed to predict in-hospital mortality.

### Covariates

We extracted patients’ age, sex, triage category, influenza type, laboratory data, baseline vital signs, underlying diseases, and outcomes from the CGRD. Laboratory data included complete blood count (CBC), white blood cell differential count, and levels of blood urea nitrogen (BUN), creatinine, aspartate transaminase (AST), alanine transaminase (ALT), C-reactive protein (CRP), sodium, potassium, and chloride. Supplementary Table 2 shows the cut-off values used for some variables in the predictive models, which were based on laboratory reference ranges and qSOFA and SIRS criteria.

### Statistical analysis

For descriptive statistics, data on continuous variables are presented as mean and standard deviation or median and interquartile ranges. Data on categorical variables are presented as counts and percentages. The results of univariate analysis exploring predictors of critical illness (for all ED patients) and mortality (for patients admitted to hospital) are expressed as odds ratios (ORs) with 95% confidence intervals (CIs) determined through simple logistic regression. We developed a two-stage modeling method to predict hospital mortality in influenza patients. In the first stage, we constructed a multiple logistic regression model to predict the outcome of critical illness for all ED influenza patients. The variables included were age, sex, vital signs, history, and CBC test results. Stepwise regression was used to identify variables associated with risk. After the first model was constructed, we calculated S1 score to predict critical illness (in-hospital cardiac arrest and ICU admission). In the second stage, we used inpatient data, S1 score, and other laboratory test values to construct a backward multiple logistic regression model. At each stage, we built a nomogram to illustrate the prediction model. The concordance index, which was obtained from the area under the receiver operating curve (AUROC), was used to assess the predictive value of the model. Statistical analysis was completed using SAS 9.4 and R-Studio. A two-tailed *p*-value of less than 0.05 was considered statistically significant.

### Validation

In order to evaluate the effect of the prediction model, validation is needed. Meanwhile, we also extracted data of Kaohsiung branch hospitals from CGRD for model validation according to the consistent inclusion/exclusion criteria. These two branches are both tertiary hospitals and totally separated in the way of patient source and geographic. Linkou branch is located in the northern part of Taiwan while Kaohsiung branch is located in the southern part of Taiwan. Therefore, the possibility of overlapping of patient source is minimum or negligible.

The data of 919 influenza patients has been compared with modeling group and described in the appendix (Supplementary Table 3). We also use the area under the curve (AUC) of a receiver operating curve (ROC) to evaluate stage I and stage II for validation prediction model.

## Results

### Patient inclusion and outcomes

A total of 1,183,266 patients visited the ED of the CGMH Linkou branch between 2010 and 2016. Within this group, 27,550 patients received a rapid diagnostic test for influenza and 6413 patients were diagnosed with influenza (Fig. 1). We chose to focus on the adult population, and therefore excluded 1782 patients who were younger than 18 years old. An additional 60 patients were excluded because the type of influenza was not recorded, and 2876 patients were excluded because CBC test data were missing. We eliminated six patients who were readmitted within 72 h to avoid duplication. Finally, we excluded one patient who died in the ED.

Consequently, the sample used in our analysis included 1680 patients. The sample was divided into two groups: patients who were discharged home and patients who were admitted to hospital. In total, 508 (30.2%) patients were admitted to the hospital, including 334 (65.7%) who were admitted to ordinary wards only and 174 (34.3%) who were transferred to the ICU. Of the patients treated in ordinary wards, 297 (89.0%) were discharged alive and 37 (11.0%) died in hospital. Of the patients transferred to the ICU, 139 (79.9%) were discharged alive and 35 (20.1%) died in hospital.

### Data extraction

We extracted the clinical information for the 1680 patients from the CGRD. The information extracted included patients’ age, sex, triage category, influenza type, triage vital signs, and underlying comorbidities. We also extracted patients’ laboratory data, including complete blood count, white blood cell differential count, and biochemistry data (Table 1). The ICU admission rate was 10.36% (174/1680) while the mortality rate was 4.29% (72/1680). In our sample, 211 patients were in critical condition and were admitted to the ICU or died in hospital. The remaining 1469 patients were considered less critical and were discharged from the ED or discharged alive after receiving only general care in ordinary wards.

**Table 1:** Baseline characteristics of 1680 in influenza patients in ED

Variable	Statistics
**Age (y/o),** Mean ± SD	51.40 ± 19.45
**Sex**, n(%)	
Female	846 (50.36)
Male	834 (49.64)
**Triage**, n(%)	
I	160 (9.52)
II	463 (27.56)
III	939 (55.89)
IV	110 (6.55)
V	8 (0.48)
**Flu type**, n(%)	
A	1311 (78.04)
B	369 (21.96)
**Baseline vital sign**, Mean ± SD	
Body temperature (^。^C)	38.14 ± 1.24
Heart Rate	107.1 ± 20.05
Respiratory Rate	20.17 ± 3.61
Systolic Blood Pressure (mmHg)	143.5 ± 30.35
Diastolic blood pressure (mmHg)	85.50 ± 35.54
Mean Arterial Pressure (mmHg)	104.8 ± 28.68
Glasgow Coma Scale: GCS < 15, n (%)	156 (9.29)
**Past History**, n(%)	
Hypertension	598 (35.60)
Diabetes Mellitus	377 (22.44)
Coronary Artery Disease	229 (13.63)
Ischemia Stroke	232 (13.81)
Peripheral Vascular Disease	28 (1.67)
ESRD	240 (14.29)
COPD	397 (23.63)
Liver cirrhosis	76 (4.52)
Intracerebral hemorrhage	58 (3.45)
Cancer	254 (15.12)
**Lab Data**, Median (IQR)	
WBC (10^3^/mm^3^)	7.2 (5.3–9.8)
RBC (10^6^/mm^3^)	4.46 (3.95–4.89)
Hb (g/dL)	13.00 (11.40–14.35)
Hct (%)	38.80 (34.60–42.50)
Platelet (fL)	179.0 (141.0–219.5)
Segment (%)	78.0 (69.8–84.3)
Band > 3%, n (%)	119 (7.08)
Creatinine (mg/dl)	0.90 (0.70–1.18)
BUN (mg/dl)	13.9 (9.4–23.3)
AST (U/L)	34.0 (25.0–57.0)
ALT (U/L)	24.0 (17.0–39.0)
CRP (mg/L)	28.56 (11.30–67.60)
Na (mmol/L)	137.0 (135.0–139.0)
K (mmol/L)	3.7 (3.4–4.1)
Cl (mmol/L)	104.0 (99.0–109.0)

The mean age was 51.40 ± 19.45 years. Fifty-two percent were women. Most patients who visited the ED were classified as triage category III (*n* = 939 [55.89%]). Most of the patients tested had influenza A (*n* = 1311 [78.04%]) and the remainder had influenza B (*n* = 369 [21.96%]). The mean body temperature measured when initial vital signs were assessed for ED triage was 38.14 °C ± 1.24, which is considered febrile. One-hundred and fifty-six patients (9.29%) scored less than 15 points on the Glasgow coma scale, implying altered mental status. The median level of C-reactive protein was 28.56 mg/L, which is above the normal range.

Univariate analysis for predicting critical care and in-hospital mortality in patients diagnosed with influenza.

The results of the univariate analysis (Table 2) indicated that elderly people (> 65 years) were at higher risk of admission to the ICU (OR = 1.7, 95% CI). Analysis by sex showed that males were more likely to require critical care than were females (OR = 1.74, 95% CI:1.30–2.34). Patients with a history of comorbidities (e.g., hypertension, diabetes mellitus, coronary artery disease, stroke, ESRD, or intracerebral hemorrhage) were at higher risk of critical illness, with ORs ranging from 1.61 to 2.53 and all *p*-values less than 0.05.

**Table 2:** Univariate analysis for predicting Critical care in patients diagnosed with influenza

Variables	Less critical (*N* = 1469)	Critical Care (*N* = 211)	OR (95%CI)	*P*-value
**Age**	50.3 ± 19.5	59.4 ± 17.3	1.02 (1.02–1.03)	< 0.001
Age < 65 y/o	1103 (75.1)	135 (64.0)	1	
Age > =65 y/o	366 (24.9)	76 (36.0)	1.70 (1.25–2.30)	< 0.001
**Sex**				
Female	765 (52.1)	81 (38.4)	1	
Male	704 (47.9)	130 (61.6)	1.74 (1.30–2.34)	< 0.001
**Triage**				
3/4/5	1031 (70.2)	26 (12.3)	1	
1/ 2	438 (29.8)	185 (87.7)	16.75 (10.94–25.63)	< 0.001
**Vital sign**				
MAP	105.6 ± 29.1	99.2 ± 24.3	0.99 (0.98–0.99)	< 0.001
MAP<=70	1433 (98.0)	189 (92.6)	1	
MAP> 70	29 (2.0)	15 (7.4)	3.92 (2.06–7.45)	< 0.001
GCS	14.8 ± 1.1	12.6 ± 3.8	0.68 (0.63–0.72)	< 0.001
GCS = 15	1391 (94.7)	133 (63.0)	1	
GCS < 15	78 (5.3)	78 (37.0)	10.46 (7.29–15.00)	< 0.001
**Past History**				
Hypertension	496 (33.8)	102 (48.3)	1.84 (1.37–2.46)	< 0.001
Diabetes Mellitus	295 (20.1)	82 (38.9)	2.53 (1.87–3.43)	< 0.001
Coronary Artery Disease	179 (12.2)	50 (23.7)	2.24 (1.57–3.19)	< 0.001
Peripheral Vascular Disease	26 (1.8)	2 (0.9)	0.53 (0.13–2.25)	0.391
Stroke	191 (13.0)	41 (19.4)	1.61 (1.11–2.34)	0.012
ESRD	186 (12.7)	54 (25.6)	2.37 (1.68–3.35)	< 0.001
COPD	340 (23.1)	57 (27.0)	1.23 (0.89–1.70)	0.217
Liver Cirrhosis	67 (4.6)	9 (4.3)	0.93 (0.46–1.90)	0.847
Intracerebral hemorrhage	45 (3.1)	13 (6.2)	2.08 (1.10–3.92)	0.024
Cancer	216 (14.7)	38 (18.0)	1.27 (0.87–1.86)	0.211
**CBC Test**				
WBC (10^3^/mm^3^)	7.8 ± 3.8	10.0 ± 6.6	1.10 (1.07–1.13)	< 0.001
WBC < 4	130 (8.8)	33 (15.6)	1	
4 < =WBC < =12	1193 (81.2)	114 (54.0)	0.38 (0.25–0.58)	< 0.001
WBC > 12	146 (9.9)	64 (30.3)	1.73 (1.07–2.80)	0.026
Segment	75.4 ± 11.9	75.8 ± 19.1	1.00 (0.99–1.01)	0.674
Segment> = 75%	885 (60.3)	148 (70.1)	1.55 (1.13–2.12**)**	0.006
Segment< 75%	583 (39.7)	63 (29.9)	1	
Band	0.5 ± 2.6	3.7 ± 7.5	1.16 (1.12–1.20)	< 0.001
Band<=3%	1408 (95.8)	153 (72.5)	1	
Band> 3%	61 (4.2)	58 (27.5)	8.75 (5.89–13.01)	< 0.001
RBC (10^6^/mm^3^)	4.4 ± 0.8	4.1 ± 0.9	0.58 (0.48–0.69)	< 0.001
Hb	12.8 ± 2.2	12.0 ± 2.7	0.86 (0.81–0.91)	< 0.001
Hb < =12 g/dL	463 (31.5)	100 (47.4)	1.96 (1.46–2.62)	< 0.001
Hb > 12 g/dL	1006 (68.5)	111 (52.6)	1	
Hct (%)	38.3 ± 6.0	35.7 ± 7.6	0.94 (0.92–0.96)	< 0.001
Platelet	184.5 ± 68.3	173.7 ± 81.8	1.00 (1.00–1.00)	0.037
Platelet> = 150 fL	1045 (71.1)	122 (57.8)	1	
Platelet< 150 fL	424 (28.9)	89 (42.2)	1.80 (1.34–2.42)	< 0.001
Observation hours	10.9 ± 7.6	10.5 ± 7.0	0.99 (0.97–1.02)	0.653

Out of 1680 patients in this study, 508 were hospitalized. Fourteen percent (72/508) died in hospital, either on the ward or in the ICU. We explored the laboratory data of hospitalized patients in more detail (Table 3). There were associations between in-hospital mortality and laboratory values, including creatinine (OR = 1.17, 95% CI:1.07–1.27), BUN (OR = 1.02, 95% CI:1.01–1.03) and CRP (OR = 1.01, 95% CI:1.001–1.010).

**Table 3:** Univariate analysis of Laboratory data for predicting in-hospital death in 508 admitted-to-ward patients

Variables	Survival to discharge (*N* = 436)	In-hospital death (*N* = 72)	OR (95%CI)	*P*-value
Cr	1.8 ± 2.1	2.9 ± 3.1	1.17 (1.07–1.27)	< 0.001
Cr < =1.2	278 (63.9)	30 (41.7)	1	
1.2 < Cr < =2	73 (16.8)	13 (18.1)	1.65 (0.82–3.32)	0.1608
2 < Cr < =3.5	34 (7.8)	12 (16.7)	3.27 (1.53–6.98)	0.0022
3.5 < Cr < =5	9 (2.1)	3 (4.2)	3.09 (0.79–12.03)	0.1041
Cr > 5	41 (9.4)	14 (19.4)	3.16 (1.55–6.46)	0.0016
BUN	25.7 ± 22.4	40.4 ± 29.9	1.02 (1.01–1.03)	< 0.001
BUN< 26	269 (69.5)	29 (40.3)	1	
BUN> = 26	118 (30.5)	43 (59.7)	3.38 (2.01–5.68)	< 0.001
AST	125.2 ± 472.1	198.0 ± 393.1	1.00 (1.00–1.00)	0.2772
AST < =136	260 (87.0)	45 (70.3)	1	
AST > 136	39 (13.0)	19 (29.7)	2.81 (1.49–5.30)	0.0014
ALT	62.5 ± 201.5	116.1 ± 458.4	1.00 (1.00–1.00)	0.1402
ALT<=144	396 (95.0)	65 (92.9)	1	
ALT> 144	21 (5.0)	5 (7.1)	1.45 (0.53–3.98)	0.4704
CRP (mg/L)	87.4 ± 88.2	142.2 ± 105.6	1.01 (1.00–1.01)	< 0.001
CRP < =50	197 (47.1)	20 (29.9)		
CRP > 50	221 (52.9)	47 (70.1)	2.09 (1.20–3.66)	0.0093
Na	137.2 ± 4.8	137.1 ± 6.1	1.00 (0.95–1.05)	0.8836
Na < =134	98 (22.7)	22 (30.6)	1	
134 < Na < =148	329 (76.2)	47 (65.3)	0.64 (0.37–1.11)	0.11
Na > 148	5 (1.2)	3 (4.2)	2.67 (0.59–12.03)	0.2002
K	3.9 ± 0.7	4.0 ± 0.8	1.16 (0.82–1.63)	0.4006
K < =3.5	134 (31.0)	26 (36.1)		
3.5 < K < =5	270 (62.5)	36 (50.0)	0.69 (0.40–1.19)	0.1775
K > 5	28 (6.5)	10 (13.9)	1.84 (0.80–4.24)	0.1523
Cl	104.4 ± 7.3	106.1 ± 7.9	1.03 (0.99–1.08)	0.1798
Cl < =98	29 (23.4)	6 (12.8)		
98 < Cl < 110	67 (54.0)	22 (46.8)	1.59 (0.58–4.32)	0.3664
Cl > =110	28 (22.6)	19 (40.4)	3.28 (1.14–9.42)	0.0273
hospital stay	18.5 ± 15.2	25.2 ± 22.8	1.02 (1.01–1.03)	0.0023

### Two-stage model for predicting in-hospital mortality in influenza patients

Multiple logistic regression analysis was used to identify risk factors for admission to critical care and in-hospital mortality. These risk factors are summarized in Table 4. In the Stage I model predicting critical illness (i.e., requiring admission to the ICU), hypothermia (versus normal temperature) appeared to be a risk factor (OR = 1.92, 95% CI: 1.04–3.56). Tachypnea (OR = 4.94, 95% CI: 3.47–7.03), lower systolic blood pressure (OR = 2.35 95% CI: 1.25–4.43), diabetes mellitus (OR = 1.87, 95% CI: 1.28–2.72), leukocytosis (OR = 2.22, 95% CI: 1.45–3.38), leukopenia (OR = 2.70, 95% CI: 1.56–4.66), and a high percentage of segmented neutrophils (OR = 2.10, 95% CI: 1.39–3.16) were also associated with admission to critical care in our analysis. Bandemia was associated with the highest odds ratio in the Stage I model (OR = 5.43, 95% CI: 3.27–9.01).

**Table 4:** Two-Stage model estimates by multiple logistic regression

Parameter/ Variable	OR(95%CI)	*P*-value
***Stage I Model: predicting critical illness in ED (n = 1680)***		
Age	1.01 (1.00–1.02)	0.118
BT (< 36 vs. 36 ~ 38)	1.92 (1.04–3.56)	0.037
BT (> = 38 vs. 36 ~ 38)	0.42 (0.29–0.61)	< 0.001
RR (> = 22 vs. < 22)	4.94 (3.47–7.03)	< 0.001
SBP (<=100 vs. > 100)	2.35 (1.25–4.43)	0.008
DM (Y vs N)	1.87 (1.28–2.72)	0.001
WBC (< 4 vs 4 ~ 12)	2.70 (1.56–4.66)	< 0.001
WBC (> 12 vs 4 ~ 12)	2.22 (1.45–3.38)	< 0.001
Segment (> = 75% vs. < 75%)	2.10 (1.39–3.16)	< 0.001
Band (> 3% vs < 3%)	5.43 (3.27–9.01)	< 0.001
***Stage II Model: predicting in-hospital mortality (n = 508)***		
BUN (mg/dl)	1.01 (1.00–1.02)	0.010
CRP	1.00 (1.00–1.01)	0.115
S1 score (Risk of critical illness) (0 ~ 100)	1.02 (1.01–1.04)	< 0.001

**Fig. 2:**
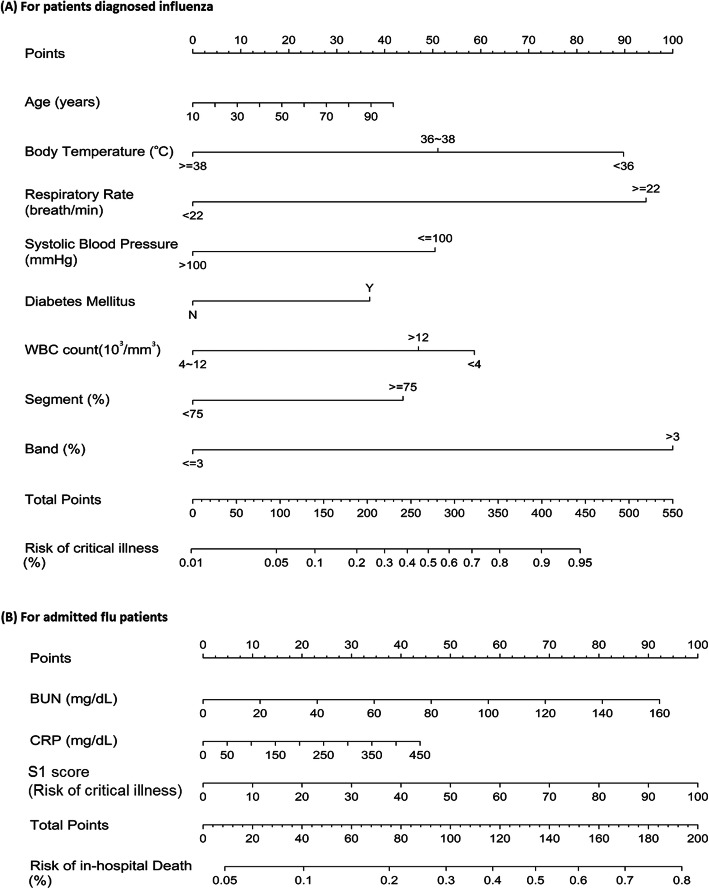
Nomogram: scoring system for predicting risk of critical illness (**a**) and in-hospital death (**b**)

In the Stage II model predicting in-hospital cardiac arrest, we used laboratory data and the Stage I model’s S1 score for critical illness to predict in-hospital mortality using multiple logistic regression. Backward selection showed that after adjustment CRP and BUN were significant risk factors for in-hospital mortality. Figure 2 shows the nomogram for the two models, allowing for rapid estimation of risk in clinical practice.

After developing our 2-stage prediction model, we used data of Kaohsiung branch hospital for validation. The AUC for the stage I model (S-I AUC) for predicting critical illness was 0.889 when using validation data. The AUC for the stage II model (S-II AUC) for predicting in-hospital mortality was 0.766 when using validation data.

## Discussion

Many scoring systems and parameters are used to predict the prognosis of sepsis patients. However, only a few focus on influenza patients. After the Third International Consensus Definitions for Sepsis and Septic Shock proposed the use of qSOFA to assess the prognosis of sepsis patients in 2016 [16], there were many studies comparing the accuracy of qSOFA and SIRS. Several studies focused on influenza patients. In general, qSOFA scores were considered superior to SIRS for predicting mortality risk in influenza patients [12, 13]. However, SIRS criteria remain useful for identifying infection and predicting prognosis. Several predictors identified in our study, including body temperature (BT), respiratory rate (RR), and white blood count (WBC), are among the clinical criteria for systemic inflammatory response syndrome (SIRS). Furthermore, systolic blood pressure is a criterion used in qSOFA. However, our predictive model showed no significant association between hyperthermia (BT > 38 °C) and the outcomes of interest. This is not compatible with SIRS criteria.

The debate on the biological significance of fever has been no consensus since the time of Hippocrates. Romanovsky and Szekely [17] proposed that fever as part of early phase syndrome of infection, which also included hyperalgesia, weakness, hypertension, motor agitation as an adaptation of infection develops in organism. The late phase syndrome, as the disease already progressed, may presents hypothermia, hypoalgesia, motor depression, normo- or hypotension. The late phase syndrome represents that energy saving strategies of organism, as a result of energy resources have been reduced by the costly early phase syndrome. Thus, hypothermia may be a sign of disease progression. However, the causative relationship between the thermoregulatory manifestations is beyond the scope of the objective of our study. A meta-analysis [18] reported that septic patients with fever estimated mortality rate was ~ 22%, which was higher (~ 31%) in normothermic patients, while it was the highest (~ 47%) in the hypothermic patients. In a retrospective study [19], if found that sepsis patients with hypothermia within 24 h is associated with increased short- (day 28) and long-term (1 year) mortality. The study also proposed that hypothermia maybe a useful prognostic factor for sepsis-induced immunosuppression. Therefore, it is reasonable to consider and explain that hypothermia is a poor prognosis factor in infection or sepsis patients.

Diabetes mellitus is thought to be associated with sepsis-related outcomes. A systematic review and meta-analysis including 234 articles and a total of 610,782 participants evaluated risk factors for severe outcomes in patients with seasonal and pandemic influenza [20]. The study reported that diabetes mellitus was associated with a higher risk of ICU admission and mortality in pandemic influenza (OR = 2.21, 95% CI: 1.37–3.58). However, for seasonal influenza, diabetes mellitus was associated only with a higher risk for hospital admission. In our model, bandemia was the strongest predictor and had the highest OR. This finding is compatible with the geriatric influenza death (GID) score developed by Chung, C.Y. et al. for predicting mortality in older people with influenza in the ED [21]. Their retrospective study used scores comprised of five independent predictors, including bandemia. As in our study, bandemia had the highest OR (OR = 7.97, 95% CI: 2.14–29.65). However, their study population was limited to people over 65 years old and the cut-off value for bandemia differed from that used in our study (3% versus 10%).

SIRS criteria and qSOFA scores have been used to predict outcomes for influenza patients in the ED. Chu, S. E et al. conducted a retrospective study that included 3561 ED patients with positive influenza tests and reported that qSOFA was a useful predictor of prognosis for influenza patients (12). When qSOFA scores were greater than or equal to three, qSOFA showed high specificity for ICU admission (sensitivity 3.2%, specificity 99.7%) and mortality (OR = 22.46, 95% CI: 4.33–116.61; sensitivity 1.3%, specificity 99.7). However, it may not be a good tool for screening and triage because of its poor sensitivity. A retrospective study conducted by Tai et al. recruited 409 geriatric patients in the ED and reported that SIRS scores greater than or equal to three showed moderate performance for prediction of mortality (OR = 3.37, 95% CI: 1.05–10.73; sensitivity 60%, specificity 70%). Other studies have challenged the value of qSOFA and SIRS criteria in predicting mortality and complications in the ED, even in sepsis patients [22, 23].

**Fig. 3:**
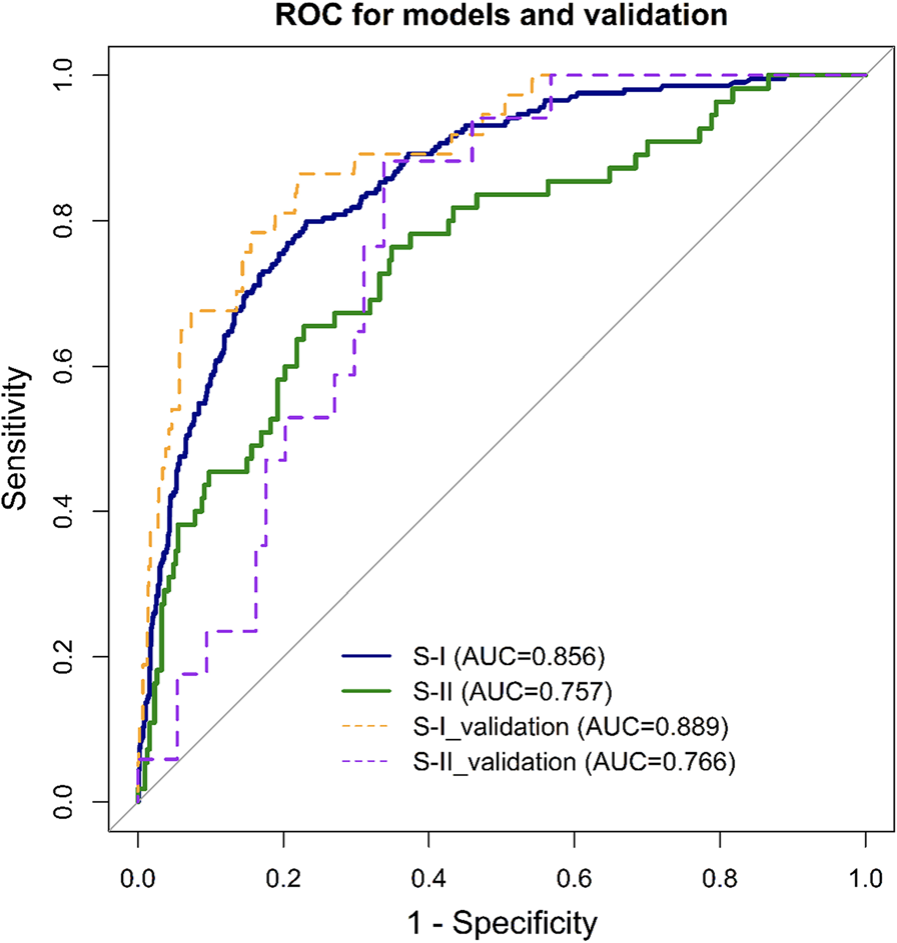
Model prediction assessment for analyzed data (Linkou branch) and validation data (the other 5 branches)

In the present study, we developed a two-stage model to predict outcomes in different patient groups. The model enabled excellent and detailed risk stratification. In the stage I model, we calculated the risk of ICU admission based on body temperature, respiration, systolic blood pressure, diabetes mellitus, and white blood cell differential count, omitting multiple serum biomarkers. In stage II, we aimed to identify admitted patients who were at higher risk of in-hospital mortality using the results of additional laboratory tests (Fig. 2). Risks could be calculated using S1 score, blood urea nitrogen, and C-reactive protein level. The model had excellent discrimination and a good fit. The receiver operating characteristic (ROC) curves for critical illness and in-hospital mortality are shown in Fig. 3. The area under the curve (AUC) for our stage I model predicting critical illness was 0.856. The AUC for the stage II model predicting in-hospital mortality was 0.757, indicating that the two-stage model had excellent discrimination.

### Limitations

Although this single-center retrospective study had a large sample size and is of significant value, it has some limitations. The Linkou branch of the CGMH is a tertiary hospital in northern Taiwan. Our validation data came from the Kaohsiung branch of the CGMH, which is another tertiary hospital in the south of Taiwan. The severity of comorbidities and the prevalence of specific diseases are significantly higher in the CGRD than in Taiwan’s general population, which may have biased our study. The model may need to be applied with caution in smaller medical settings. However, our data came from the CGRD (168,000 cases visiting the ED per year); thus, our model is still strongly predictive of critical care admission and in-hospital mortality.

## Conclusion

The two-stage model is an efficient and excellent risk-stratification tool for predicting critical illness and mortality. The model may be an optional tool other than qSOFA and SIRS criteria for helping clinical physicians with the disposition of ED patients with influenza.

## Supplementary Information

**Additional file 1: Supplementary Table 1.** ICD9/10 of disease.

**Additional file 2: Supplementary Table 2. **Cut-off values and references of the data.

**Additional file 3: Supplementary Table 3.** Information from validation data compared with modeling data.

## Data Availability

The datasets used and analyzed during the current study are available from the corresponding author on reasonable request.

## Abbreviations

ALT: Alanine transaminase
AST: Aspartate transaminase
AUC: Area under the curve
AUROC: Area under the receiver operating characteristic curve
BT: Body temperature
BUN: Blood urea nitrogen
CBC: Complete blood count
CGMH: Chang Gung Memorial Hospital
CGRD: Chang Gung Research Database
CI: Confidence intervals
CRP: C-reactive protein
EDs: Emergency departments
ORs: Odds ratios
qSOFA: Quick Sequential Organ Failure Assessment
ROC: Receiver operating characteristic
RR: Respiratory rate
SIRS: Systemic inflammatory response syndrome
WBC: White blood count
