# Supplementary material for: Two-stage prediction model for in-hospital mortality of patients with influenza infection
Source: BMC Infect Dis. 2021 May 19;21:451. doi: 10.1186/s12879-021-06169-6 (PMC8131882; doi:10.1186/s12879-021-06169-6)
Supplement: Supplementary file 1 — Additional file 1: Supplementary Table 1. ICD9/10 of disease. [file 12879_2021_6169_MOESM1_ESM.docx]

**Supplementary table 1** ICD9/10 of disease

| **Disease** | **ICD9** | **ICD10** |
| --- | --- | --- |
| Hypertension (HTN) | 401 402 | I10 I11 |
| Diabetes Mellitus (DM) | 250 | E10 E11 |
| Coronary artery disease (CAD) | 414 429 | I25 I24 |
| Vascular Dz (VD) | 440 | I700 I702 I703 I704 I705 I706 I707 I708 I709 |
| Intracerebral hemorrhage (ICH) | 430 431 432 | I60 I61 I62 |
| Stroke | 433 434 435 436 437 438 | I63 I64 I65 I66 I67 I68 I69 |
| ESRD | 585 | N17 N18 N19 |
| COPD | 490 491 492 493 494 495 496 | J440 J449 J441 |
| Liver Cirrhosis (LC) | 5712 5715 5716 | K703 K743 K744 K745 K746 |
| Cancer | 140~209 | C00 C01~C80 |
| Influenza | 487 488 | J09 J10 J11 |
