# Supplementary material for: Two-stage prediction model for in-hospital mortality of patients with influenza infection
Source: BMC Infect Dis. 2021 May 19;21:451. doi: 10.1186/s12879-021-06169-6 (PMC8131882; doi:10.1186/s12879-021-06169-6)
Supplement: Supplementary file 2 — Additional file 2: Supplementary Table 2. Cut-off values and references of the data. [file 12879_2021_6169_MOESM2_ESM.docx]

**Supplementary table 2**

| **Item** | **unit** | **Cut-off** | **Reference** |
| --- | --- | --- | --- |
| Body Temperature (BT) | ^。^C | 36, 38 | SIRS |
| Heart Rate (HR) | /1 min | 90 | SIRS |
| Respiratory Rate (RR) | /1 min | 22 (20) | qSOFA (SIRS) |
| Systolic blood pressure (SBP) | mmHg | 100 | qSOFA |
| Mean Arterial pressure (MAP) | mmHg | 70 |  |
| Glasgow Comma Scale (GCS) | Score | 15 | qSOFA |
| Creatinine (Cr) | mg/dL | 1.2, 2, 3.5, 5 | Normal range cut-off |
| Blood urea nitrogen (BUN) | mg/dL | 26 | Normal range cut-off |
| AST | (U/L) | 136 | 3-fold upper limit |
| ALT | (U/L) | 144 | 3-fold upper limit |
| CRP | (mg/L) | 50 | Normal range cut-off |
| WBC | 10^3^/uL | 4, 12 | SIRS |
| Platelet | 10^3^/uL | 150 | Normal range cut-off |
| Hemoglobin (Hb) | g/dL | 12 | Normal range cut-off |
| Sodium (Na) | mmol/L | 134, 148 | Normal range cut-off |
| Potassium (K) | mmol/L | 3.5, 5 | Normal range cut-off |
| Chloride (Cl) | mmol/L | 98, 110 | Normal range cut-off |
| Segment | % | 75 | Normal range cut-off |
| Band | % | 3 | Normal range cut-off |
