# Supplementary material for: Two-stage prediction model for in-hospital mortality of patients with influenza infection
Source: BMC Infect Dis. 2021 May 19;21:451. doi: 10.1186/s12879-021-06169-6 (PMC8131882; doi:10.1186/s12879-021-06169-6)
Supplement: Supplementary file 3 — Additional file 3: Supplementary Table 3. Information from validation data compared with modeling data. [file 12879_2021_6169_MOESM3_ESM.docx]

**Supplementary table 3** Information from validation data compared with modeling data

|  | Modelling data | Validation data |
| --- | --- | --- |
| **Variable** | **Linkou** | **Kaohsiung** |
| Number of cases | 1680 | 919 |
| Age (y/o) | 51.40 ± 19.45 | 49.63± 18.29 |
| Sex=M, n(%) | 834 (49.64) | 407 (45.17) |
| Flu type, n(%) |  |  |
| A | 1311 (78.04) | 708 (78.58) |
| B | 369 (21.96) | 193 (21.42) |
| Body Temperature (C) | 38.14 ± 1.24 | 38.22 ± 1.60 |
| Respiratory Rate | 20.17 ± 3.61 | 19.12 ± 3.20 |
| SBP (mmHg) | 143.5 ± 30.35 | 146.33 ± 32.12 |
| Past history w/ DM | 377 (22.44) | 143 (15.87) |
| BUN (mg/dl) | 13.9 (9.4 - 23.3) | 11 (8.00 -16.00) |
| CRP (mg/L) | 28.56 (11.3 - 67.6) | 17.65 (7.80 -37.80) |
| WBC (10^3^/mm^3^) | 7.2 (5.3 - 9.8) | 6.5 (5.20 -8.30) |
| Segment (%) | 78.0 (69.8 - 84.3) | 72 (59.00 -83.05) |
| Band >3% , n(%) | 119 (7.08) | 6 (0.67) |
| Admission | 508 (30.24) | 129 (14.32) |
| ICU critical care | 174 (10.36) | 36 (4.00) |
| Death | 72 (4.29) | 21 (2.33) |
| S-I AUROC | 0.856 | 0.889 |
| S-II AUROC | 0.757 | 0.766 |
